# Socioeconomic disparities in access to intensive insulin regimens for adults with type 1 diabetes: a qualitative study of patient and healthcare professional perspectives

**DOI:** 10.1186/s12939-019-1061-8

**Published:** 2019-10-11

**Authors:** Anne Scott, Alicia O’Cathain, Elizabeth Goyder

**Affiliations:** School of Health and Related Research, University of Sheffield, Sheffield, S1 4DA UK

**Keywords:** Type 1 diabetes mellitus, Socioeconomic status, Socioeconomic inequalities, Healthcare access, Health literacy, Healthcare disparities

## Abstract

**Background:**

Type 1 diabetes is a complex chronic condition which requires lifelong treatment with insulin. Health outcomes are dependent on ability to self-manage the condition. Socioeconomic inequalities have been demonstrated in access to treatment and health outcomes for adults with type 1 diabetes; however, there is a paucity of research exploring how these disparities occur. This study explores the influence of socioeconomic factors in gaining access to intensive insulin regimens for adults with type 1 diabetes.

**Methods:**

We undertook a qualitative descriptive study informed by a phenomenological perspective. In-depth face-to-face interviews were conducted with 28 patients and 6 healthcare professionals involved in their care. The interviews were analysed using a thematic approach. The Candidacy theory for access to healthcare for vulnerable groups framed the analysis.

**Results:**

Access to intensive insulin regimens was through hospital-based specialist services in this sample. Patients from lower socioeconomic groups had difficulty accessing hospital-based services if they were in low paid work and because they lacked the ability to navigate the healthcare system. Once these patients were in the specialist system, access to intensive insulin regimens was limited by non-alignment with healthcare professional goals, poor health literacy, psychosocial problems and poor quality communication. These factors could also affect access to structured diabetes education which itself improved access to intensive insulin regimens. Contact with diabetes specialist nurses and attendance at structured diabetes education courses could ameliorate these barriers.

**Conclusions:**

Access to intensive insulin regimens was hindered for people in lower socioeconomic groups by a complex mix of factors relating to the permeability of specialist services, ability to navigate the healthcare system and patient interactions with healthcare providers. Improving access to diabetes specialist nurses and structured diabetes education for vulnerable patients could lessen socioeconomic disparities in both access to services and health outcomes.

## Background

### The management of type 1 diabetes

Type 1 diabetes is a chronic disease in which self-care is critical to successful outcomes [1]. In England the care of individuals with type 1 diabetes is set through the implementation of quality standards published by the National Institute for Health and Care Excellence (NICE) and monitored by the National Diabetes Audit. These standards comprise care processes designed to monitor the progression of the disease. NICE guidance does not specify attendance at any particular centre for diabetes care but does stipulate that individuals with type 1 diabetes should receive care from a multi-disciplinary team working together to provide consistency of advice. In addition, the guidance recommends that all people over the age of 12 should be offered attendance at a Structured Education Programme shortly after diagnosis.

There is variation in diabetes services provision within England. Some may be led by GP practices, some by specialist hospital departments and some by intermediate community services [2]. Whereas individuals with type 2 diabetes tend to receive all their care within the GP surgery, individuals with type 1 diabetes may attend appointments at specialist services, the GP surgery, a mixture of both or none at all. Hence patients who only attend specialist services may not receive all the care processes carried out as part of the GP surgery remit covered under the Quality and Outcomes Framework [3]. Most individuals with type 1 diabetes tend to receive care at a specialist centre but it is estimated that up to 20% of individuals may not [3].

Central to the management of type 1 diabetes is control of blood glucose levels. Adults are advised to maintain their haemoglobin A1c (HbA1c) within a range consistent with minimising the long term complications of diabetes [4]. Technology supporting diabetes self-management comprises methods of insulin delivery and equipment to check blood glucose levels.

Intensive insulin regimens (IIRs) involve multiple daily injections (MDI) in conjunction with carbohydrate counting or insulin pump therapy (continuous subcutaneous insulin infusion (CSII)).

The National Institute for Health and Care Excellence (NICE) recommends that individuals diagnosed with type 1 diabetes should be offered MDI basal-bolus regimens as the treatment of choice [4]. MDI provides a low level of insulin in an effort to mimic a normal pancreas. In addition, short action bolus injections are given in order to lower blood glucose levels after a meal. CSII are portable pumps designed to infuse insulin via an implanted cannula in such a way as to mimic insulin delivery. NICE recommends restricted use of CSII for individuals with type 1 diabetes. In England adults with type 1 diabetes are offered CSII when efforts to achieve target haemoglobin A1c (HbA1c) levels using MDIs cannot be achieved without resultant disabling hypoglycaemia or when HbA1c remains high on this regimen despite high levels of self-care [5].

### Socioeconomic disparities in type 1 diabetes

Socioeconomic disparities relate to the systematic differences in health outcomes experienced by the affluent in society compared with the less well-off [6–8]. Despite being acknowledged as high profile targets of health care policy, socioeconomic inequalities have proved consistently difficult to eliminate in England’s NHS [9]. Although effective management of diabetes offers better health outcomes in terms of minimising the risks of short-term and long-term complications [10], a recent review of adults with type 1 diabetes found associations between socioeconomic status (SES) and disparities in mortality, morbidity and diabetes management [11]. In terms of diabetes management, higher SES is associated with attendance at specialist diabetes services [12–14] and the evidence suggests that those attending specialist services are more likely to have received diabetes education and to have lower HbA1c levels [14]. Individuals with higher SES inject insulin more frequently on a daily basis, are better informed about diabetes management and more of them attend structured education [12]. Individuals with lower SES are less likely to adopt IIRs which is important because more intensive regimens are associated with better outcomes [10].

Three ways in which socioeconomic position may influence health outcomes in diabetes posited by Brown et al. [15] comprise: access to care; process of care; and individual behaviour. In the conceptual framework proposed by the authors, access to care includes visits to both primary and specialist services. Process of care comprises aspects of diabetes care monitoring including: HbA1c levels; eye checks; cholesterol levels and foot checks. Individual behaviour relates to the work that people with diabetes must accomplish in order to manage the condition. The authors discuss the respective roles played by providers of healthcare, healthcare system characteristics and the community in which individuals reside. It is suggested that poor health outcomes are a combination of lack of access to high quality health care resulting in inadequate and inferior treatment (resulting in increased morbidity) and deficits in self-care behaviour [15].

Access to healthcare is one influence amongst many other determinants of health outcomes [9] and has the potential to enable treatment and improve health [16]. Preventive healthcare is key to minimising the onset of diabetes complications [17] and successful partnerships between patients and healthcare professionals are essential if improvements in health outcomes are to be achieved for people with diabetes [18]. Equity of access is particularly important in terms of diabetes care since it is known that the complications associated with poor diabetes management may be prevented.

Although socioeconomic disparities are known to persist in relation to access to treatment and health outcomes for adults with type 1 diabetes, little research has explored the pathways involved in this lack of access. CSII is more expensive than MDI and is subject to restrictions on allocation in the National Health Service (NHS) in the UK [5]. Prevalence of use is well below the estimations of individuals who are likely to benefit from CSII levels (15–20%) [19]. Issues relating to the allocation of CSII in the UK may be compounded by socioeconomic disparities in access to IIRs. Some quantitative research has been conducted in the USA investigating socioeconomic disparities in access to CSII for children [20, 21]; however, there is a lack of research focusing on adults and none has been conducted in the UK with a focus on socioeconomic factors.

### Theories of access to healthcare

There is a considerable body of literature exploring the concept of access to healthcare spanning various disciplines and approaches [22] including amongst others: epidemiological, case studies and case reports; evaluative, trial, descriptive, sociological, psychological, management, and economic [23]. The literature is diverse and complex. In terms of investigating the utilisation of healthcare services one of the most important and well cited theories is the Andersen Behavioral Model of Health Services Use [24]. The original model was first developed in the 1960s and has undergone a number of iterations. The model has been used extensively in studies of utilisation predominantly adopting a quantitative approach [25].

However, focusing on the utilisation of services (realised access) does not necessarily provide insights into the inequities relating to potential access and simply observing disparities in utilisation does not elucidate the complex intricacies of supply and demand factors influencing these patterns of consumption [26]. Access to treatment may be conceptualised as a continuum with opportunities to interact with services at a number of stages [27]. At each stage individuals may choose whether or not to engage with the services on offer. Equality of treatment arises out of and is affected by an interaction between supply (healthcare provider) and demand (patient) factors.

An alternative model defines the concept of access as the ‘degree of fit’ between consumers of healthcare services and the provision of services [28]. In this model developed by Penchansky and Thomas, access is optimised by paying attention to the following dimensions: availability of services; accessibility of services; accommodation of services; affordability and acceptability.

The importance of examining both the demand and the supply side of access to healthcare has particular saliency with regard to access to healthcare by individuals in lower socioeconomic groups. It is well known that research into equity of access for individuals of low socioeconomic status is fraught with complexity [27]. For example there appears to be greater utilisation of primary care and less of some secondary care services amongst individuals in low socioeconomic groups [26, 27, 29]. Emergency services are utilised more and specialist services less by individuals in lower socioeconomic groups [26].

### Candidacy theory

Starting from a position that acknowledges the limitations of research focusing on utilisation, Dixon-Woods et al. [29] suggest that research focusing on utilisation of services consumed by individuals takes no account of factors that may have considerable influence over access. Using an approach grounded in meta-ethnography Dixon-Woods et al. [29] provide a synthesis of the literature on access to healthcare by vulnerable groups in which the concept of ‘candidacy’ is proposed as a way to understand the barriers to healthcare experienced by these individuals. The theory was developed in order to explain how the combined influences of supply and demand factors impact on the ability of vulnerable groups to access healthcare [23]. Candidacy theory captures the concept that eligibility for access to healthcare services is a jointly negotiated undertaking involving a dynamic component in which interactions between patients and healthcare providers are in a constant state of change. The key concepts (permeability, navigation, appearances, adjudication, offers and resistance) describe the various points along the patient journey through which negotiation takes place. Permeability is a term used to describe how easily individuals may access services. For example, accessing specialist services is less permeable than visiting a local general practice because greater effort is required in order to access the former. Navigation refers to the ability of individuals to negotiate healthcare services. The concept of appearances refer to the notion that individuals present themselves to healthcare services and may or may not be able to assert their candidacy for a particular treatment or service. Adjudication refers to the way in which healthcare professionals make judgements about whether or not individuals are suitable candidates for treatment. The concept of offers and acceptance illuminates aspects of non-utilisation of services in that for a variety of reasons some individuals choose either to delay or refuse treatment. The theme running through the candidacy theory concepts is that individuals in lower socioeconomic groups are disadvantaged in a number of ways including: how permeable they find services, their lack of knowledge of services and their beliefs about seeking help; their ability to present and be seen as a suitable candidate for treatment and finally whether or not offers of treatment are taken up. The theory has been used successfully to elucidate aspects of access to mental health services [30, 31] and emergency and urgent care [32, 33].

Although quantitative research has identified that socioeconomic inequity persists in terms of usage of IIRs, it has so far not yet elucidated the possible pathways involved in this inequity [11]. Qualitative research has the potential to elicit some of the key influencing factors involved. The aim of this study, therefore, was to understand how socioeconomic disparities in access to CSII and MDI might occur and this was accomplished by exploring, through qualitative interviews, patient and healthcare perspectives on accessing these regimens.

## Methods

### Study design, sample and recruitment

We undertook a qualitative descriptive study [34–36] informed by a phenomenological perspective. Phenomenological approaches aim to capture ‘how people experience some phenomenon – how they perceive it, describe it, feel about it, judge it, remember it, make sense of it and talk about it with others’ [37]. The findings of qualitative descriptive studies have considerable potential to translate into improvements in healthcare for vulnerable groups [38].

The study design was underpinned by subtle realism, a credible philosophical stance in healthcare research [39]. Subtle realism acknowledges that social reality can be studied; however, this is only possible via the interpretations of individuals and in addition the further construal of these interpretations by the researcher [40]. Although the aim was to provide a description of the experiences, events and processes involved in access [38] it was also important to go beyond description in order to provide accessible research findings with the potential to inform policy making and practice.

During April and December 2012 in-depth face to face interviews were conducted with healthcare professionals and patients in order to explore factors influencing equity in gaining access to IIRs. The sample was drawn predominantly from a hospital in central England serving a population of 300,000 individuals. The area includes a district ranked amongst the top 10% most deprived in England. Hospital-based services were chosen because type 1 diabetes care was offered within this setting, with GPs expected to refer patients requiring specialist input. A small sample of patients and healthcare professionals were also recruited from a GP practice in an area of deprivation for two key reasons. First, research shows that these areas will be more likely to encounter individuals not attending specialist centres and second, access to secondary care was through primary care and hence it was important to consider this pathway as part of the study. Patients were eligible to participate in the study if they had had type 1 diabetes for at least 1 year, were over 18 years of age and were able to speak English.

Commonly used measures of socioeconomic position include income, education and occupation. However, there is no single measure of socioeconomic position that can be comprehensively applied to all studies [41]. In the current study participants were classified using the National Statistics Socioeconomic Classification (NS_SEC) which has been adopted as the measure of socio-economic position in official statistics in the UK since 2001 [42] and in order to provide further context, by the Index of Multiple Deprivation (IMD) 2010 [43]. For the latter, individuals were classified using their postcodes and allocated a single score. It is usual to report these scores in quintiles.

### Data collection

In-depth interviews, scheduled to last approximately 1 h, were conducted with patients and healthcare professionals. Participants were given written and verbal information about the study prior to consent. Written consent was obtained from all participants who agreed to the recording of interviews and the publication of anonymised quotes. All personal data collected during the course of the study was treated as confidential. All of the transcriptions were anonymised and only anonymised transcripts were shared with academic supervisors. All data was held in password protected files.

Most patients opted for an interview at home. A strategy of purposively sampling a range of participants to take account of sociodemographic and clinical factors was adopted. Between 15 and 30 interviews were planned for patient participants and between 5 and 10 interviews were planned with healthcare professionals.

In order to ensure the quality of interviews and the data, detailed records were kept throughout the research process [44]. To ensure accuracy of the data, interviews were recorded and transcribed verbatim. Consistency of interview data was facilitated through the use of a topic guide. This provided a systematic approach whilst still allowing for spontaneity in questioning [37]. Interviews commenced with a subject which was straightforward and allowed a mainly descriptive response [37] by asking individuals to give a brief history of their condition since diagnosis. The remaining topic areas built on this initial discussion and included key treatment decisions. Healthcare professionals were questioned about the main influences on access to IIRs.

### Analysis

Interview data were analysed using a thematic approach. Thematic analysis was chosen for its potential to facilitate a rich and insightful exposition of the data [45]. Computer assisted qualitative data analysis software (NVivo™ 9) was used to facilitate the storage and retrieval of verbatim transcripts. Transcripts were coded on the basis of comparing within and between cases [46]. Particular attention was paid to differing patient characteristics during cross-comparisons. Themes were developed inductively by retrieving coded data extracts from NVivo™ 9 and were written up by the first author. Through an iterative process of writing up and discussion between the authors the list of themes was reduced and refined. During the final stages of analysis it was observed that one of the key theories of access in relation to vulnerable groups had particular saliency with the data. Themes were therefore reorganised and reframed using the key features of Candidacy theory [29].

## Results

### Description of sample

Interviews were conducted with 28 patients with type 1 diabetes and 6 healthcare professionals involved in their care. Of these participants three patients and two healthcare professionals were recruited at a GP surgery. Interviews continued with patients until saturation of themes was established and we were satisfied that a reasonably diverse sample had been achieved. This type of data saturation could not be applied to healthcare professionals because only a few delivered this type of care to patients in this hospital. We stopped interviewing healthcare professionals when we had interviewed those delivering intensive regimens. Fourteen interviews with patients were between 50 and 70 min in length; 11 were longer than 70 min and 3 were less than 50 min. Interviews with healthcare professionals lasted between 32 and 78 min with most over 45 min. The majority of patients and healthcare professionals were recruited from a specialist diabetes service in a hospital. Two primary care professionals (a practice nurse and GP) were recruited. The other healthcare professionals comprised a consultant diabetologist in the CSII clinic, a consultant diabetologist in the general diabetes clinic, a diabetes specialist nurse and a diabetes specialist dietitian. The patient sample represented a diverse group of adults (13 men and 15 women) aged between 20 and 79 (Table 1) from various socioeconomic groups.

**Table 1:** Sociodemographic characteristics of patients in the sample

Characteristics	Number
Gender	
Male	13
Female	15
Age	
18–29	4
30–39	7
40–49	10
50–59	4
60–69	2
≥ 70	1
Socioeconomic classification^a^	
Higher managerial, administrative and professional	11
Intermediate	7
Routine and manual	9
Student	1
Education	
Left school at 16 (no further qualifications)	10
Continued with education/qualifications post 16	18
Deprivation (IMD 2010)^b^	
Quintile 1 (Most deprived)	11
Quintile 2	6
Quintile 3	2
Quintile 4	6
Quintile 5 (Least Deprived)	3

^a^The Standard Occupational Classification 2010 (three classes version). Unemployed individuals were coded to their last occupation

^b^The Index of Multiple Deprivation 2010 [43].

Seventeen patients were employed, 4 patients were unemployed (3 in receipt of disability payments due to diabetes), 3 patients had retired and 3 were caring for children. Almost twice as many individuals (18) pursued a qualification post 16 years of age compared with those leaving school at 16 without qualifications (10). Higher managerial, administrative or professional individuals and intermediate classes accounted for 18 participants. There were 9 patients in lower socioeconomic groups.

Users of both types of IIR (CSII and MDI) were represented across age and gender groups. Eighteen patients were using CSII, 8 patients were on MDI involving carbohydrate counting and 2 patients were on MDI not involving carbohydrate counting (Table 2).

**Table 2:** Clinical characteristics of patients in the sample

Characteristics	Number
Diabetes duration (years)	
1–5	3
6–10	3
11–15	5
16–20	4
≥ 21	13
Age at diabetes onset	
0–10	5
11–20	8
21–30	8
31–40	5
≥ 41	2
Treatment at time of study	
CSII	18
Multiple daily injections (carbohydrate counting)	8
Basal bolus (not involving carbohydrate counting)	2
HbA1c	
≤ 7.5% (≤58 mmol/mol)	10
7.6 to 9.9% (60 mmol/mol to 85 mmol/mol)	15
≥ 10.0% (≥86 mmol/mol)	3
Complications arising from diabetes	
Reported at least one complication	16
None reported	12

### Overview of themes

The findings are presented under four main themes which align with the Candidacy theory concepts of Permeability, Navigation, Adjudication and Offers and Resistance. Although the concepts within Candidacy theory are dynamic and overlapping in nature, the themes presented here follow the sequence of a patient’s journey through healthcare pathways.

### Permeability: the ease of accessing the hospital-based specialist service

More work is required by patients to access less permeable healthcare services. Some patients are not well equipped in terms of resources and personal circumstances to successfully engage with less permeable services. Patients in lower socioeconomic groups placed emphasis on barriers to accessing services including work related factors, lifestyle and transport issues which they described as impeding their access to hospital-based services. Some patients reported that they were not paid if they took time off work and this discouraged their attendance at the hospital. For example, Patient 1, who was using CSII at the time of the interview, described how for many years work would take precedence over appointments at the hospital for financial reasons:

*I used to go to the diabetic nurses but I didn’t used to go [to] the doctor. Used to say right have an appointment to go to and then cos of work with me working on the farm and timescales and not wanting to give up work I kept putting them off and putting them off and I’d go to one or two but not go to them all... I wanted the money because obviously some of the time of year you don’t work*

*(Patient 1 – CSII – Routine and manual (unemployed))*

Healthcare professionals from both the GP surgery and the Specialist Services also described factors relating to work commitments as a hindrance to patients being able to attend either specialist services or the Structured Education Programme.

*So I think a lot of it is to do with sort of work commitments.... and of course we’re in a recession. The last thing people want to do is put any jobs at risk. They don’t want to give their employers any excuse so it may be tied in with job security as well.*

*(Healthcare Professional 1 (Specialist Services))*

Healthcare Professional 5 (Primary Care), who worked in a surgery within an area of deprivation, described how ‘chaotic’ lifestyles could lead some of her population to miss hospital appointments. Non-attendance at specialist services appeared to have serious implications beyond the missed opportunity to consult with healthcare professionals. If they missed appointments the hospital-based specialist healthcare professionals discharged them, thus limiting their access to IIRs. A number of factors conspired to make attendance at hospital less likely for this population. For example, the area has a large number of social houses and high levels of population movement. As Healthcare Professional 5 (Primary Care) reflected there appeared not to be a good ‘fit’ between the services on offer and her patients’ characteristics.

*I think a lot of our patients have really ‘chaotic’ lifestyles and that just doesn’t fit very well with regular reviews at the hospital and so a lot just they move, they change their mobile phone numbers, they just lose contact with the hospital and particularly now the hospital very quickly discharges anyone who doesn’t turn up. So then they get discharged and then if they do need to be seen at the hospital there needs to be a GP referral to refer them.*

*(Healthcare Professional 5 (Primary Care))*

Patients who were unemployed or in low paid work and were not car owners also described finding travel to the hospital difficult. Several patients reported travel barriers. When faced with having to take a number of buses to the hospital, one described preferring to seek care closer to home in his general practice.

*I’ll probably have it done at (GP surgery) just cos I don’t have to get up here (the hospital). So its two buses to get here from where I live.*

*Patient 2 – MDI – Routine and Manual (Carer)*

### Navigation: the role of health literacy in negotiating pathways through the healthcare system

Some patients appeared to be able to navigate the hospital-based specialist services more effectively than others. These patients placed a high value on gaining access to specialist expertise and expressed a preference for these services. They demonstrated an awareness of these services and knowledge about how to navigate the system whilst other participants did not. For example Patient 3 gave an account of her awareness that an insulin pump was, potentially, an option and her understanding that attending the Structured Education Programme was a requirement for eligibility. There appeared to be a proactive approach to the way in which this patient sought information in order to make a decision between an insulin pump or multiple daily injections regimen.

*I've become aware about the insulin pump that many people are now on because I wondered if I would go that way. So I made some enquiries of my own … And I was aware that I think you have to have done the course or something like it before you can go onto this anyway.*

*Patient 3 – MDI – (Higher managerial, administrative and professional)*

These patients were more successful than others at gaining access to specialist expertise which appeared to be an important prerequisite of gaining access to IIRs. Patient knowledge was important for independent navigation through specialist services, gained from regular attendance at specialist services, being a healthcare worker or having a friend or family member who was a healthcare professional. Knowing someone who had successfully navigated the system also appeared to be an advantage. A newly diagnosed patient who learned about CSII from an acquaintance and decided that her current regimen was unsatisfactory described how she was able to navigate the system herself:

*He (the family acquaintance) couldn’t believe that I'd been sent home [ … ] after he’d been and spoke to me I rang the hospital and asked if I could be put on a carbohydrate counting course so I could get used to how you count carbs and how you do have different doses of insulin every day. So I went on the course.*

*(Patient 4 – CSII – student)*

The majority of patients in the sample who actively sought information and care were in higher socioeconomic groups. They showed strong evidence of what might be described as health literacy or social capital. For example, the charity Diabetes UK was mentioned as a key source of information by these patients whilst none of the patients with lower socioeconomic status mentioned actively seeking information from this source. Healthcare professionals also reported observing high levels of health literacy in patients with higher socioeconomic status and linked this with providing some patients with an advantage in terms of gaining access to CSII:

*I think that’s right across the board whether it’s pump therapy or not you will get professional people making sure that they get the best out of the system because they know how the system works and they have the ability to use the phones and the internet and the computers and get where they want to be.*

*(Healthcare Professional 1 (Specialist Services))*

In comparison, patients in lower socioeconomic groups displayed low levels of health literacy, describing a lack of awareness of other regimens prior to their engagement with specialist services. For example, Patient 1, who was unemployed at the time of the study, received a referral to specialist services for the local Structured Education Programme. Prior to this referral he was unaware that there was a course available to help him manage diabetes.

### Presentation and adjudication: the role of patient-provider alignment in healthcare interactions

Healthcare professionals play an important role in determining who gains access to IIRs and particularly to CSII. It appeared that a complex mix of factors influenced the decision to offer the treatment to some patients. Some of these factors appeared to disadvantage individuals with poorer personal resources and lower health literacy. Whereas a good ‘fit’ between what was offered by healthcare professionals and what was sought by patients appeared to facilitate access to IIRs, conversely a poor ‘fit’ between the patient and healthcare professionals seemed to act as a barrier to accessing these services.

#### Influencing professional judgments

Healthcare professionals described decision-making around patient candidacy for CSII primarily in terms of clinical need. Difficulties in achieving acceptable glycaemic control despite attempts at following an MDI regimen were described as a major consideration, whereas a patient’s request for CSII for lifestyle reasons was described as unlikely to succeed:

*There usually needs to be some sort of clinical indication. So just if a patient happens to be type 1 but is well controlled for them just coming and saying I want a pump isn't enough.*

*(Healthcare Professional 1 (Specialist Services))*

Other factors which healthcare professionals described as influencing decision-making appeared to be more subjective such as assessing patient motivation, perceptions of patient ability to adopt a more demanding regimen and patient knowledge. These attributes were viewed by healthcare professionals as essential to ensure that patients were able to manage CSII safely. Patients who displayed an interest in managing their condition appeared to be viewed as potential candidates for IIRs by healthcare professionals in this sample:

*We’re looking for people who are motivated; who can self-care because it’s a technology that a patient will have to take ownership of [...] what indicates to me that they're self-motivated; it’s just people who are engaged in the consultation [...] someone who has an interest in self-managing a condition and who has a good knowledge of their diabetes. I think those are the main issues.*

*(Healthcare Professional 3 (Specialist Services))*

Patients in higher socioeconomic groups described taking a proactive approach to decision-making with healthcare professionals, including pushing for CSII. They described an ability to assimilate information about CSII and its potential benefits. It appeared that being able to communicate effectively was important and an ability to align culturally with healthcare professionals was influential:

*I mean I’m always quite direct with doctors and nurses and of course I speak their language.*

*(Patient 5 – CSII – Higher managerial, administrative and professional)*

Although healthcare professionals suggested that clinical need was the most important justification for CSII it seemed that some patients in higher socioeconomic groups were able to persuade healthcare professionals that they were candidates for the regimen without necessarily fulfilling this criterion. The characteristics of patients who appeared to assert themselves in consultations were encapsulated in the following account by Healthcare Professional 6 (Specialist Services). In the opinion of this member of staff patients requesting an insulin pump tended to be characterised by possessing a high level of education and familiarity with different regimens, either through an organisation such as Diabetes UK or by finding out about services through acquaintances or family.

*In terms of who would be more likely to ask about insulin pump therapy, I think a lot of people ask about therapies that they're aware that their friends or people they know. Others are very highly educated and they're more aware of what is available for them. Some of them they’re sort of involved in Diabetes UK or they’re members and therefore they’re more aware.*

*(Healthcare Professional 3 (Specialist Services))*

#### Communication in consultations

Patients in higher socioeconomic groups described high levels of involvement in consultations. In contrast, patients in lower socioeconomic groups, particularly those with poor metabolic control, described some of their interactions with healthcare professionals as an unequal partnership. For example, patients in lower socioeconomic groups who described low motivation in relation to diabetes management and reported that they did not always follow clinical recommendations also described some healthcare professionals taking a judgmental stance towards them. Some perceived that healthcare professionals seemed disinterested in them and others reported a feeling that that they had been coerced into following particular treatment regimens. Patient 6 (MDI) below had difficulty injecting himself and admitted he did not follow the advice given by healthcare professionals. He described his doctor’s communication as directive in style and reported that this led to him opting out of these consultations.

*I think what it is like he used to shout at me. Obviously I did wrong not injecting for me snacks and everything else but he didn’t shout, he just raised his voice a little […] that’s why my sugar’s obviously went high and he just gave me a lecture basically not nasty or ‘owt said if you don’t do that I could go into a coma.*

*(Patient 6 – MDI – Routine and manual – unemployed)*

Some patients in lower socioeconomic groups with poor metabolic control also stressed the importance of being ‘heard’ in the consultation and expressed a hope that healthcare professionals would listen to their difficulties in managing the regimen. This aspect of communication mentioned predominantly by patients in lower socioeconomic groups is explored further in the final theme:

*I know where he's coming from because I do listen to what he was saying but the other thing is I think he needs to listen to me more about how I'm feeling what I've been undertaking.*

*(Patient 7 – MDI – Routine and manual – unemployed)*

### Offers, resistance and acceptance: aspects of the service ameliorating socioeconomic disparities

#### Diabetes specialist nurses provide a more accessible service

Some patients from lower socioeconomic groups described resisting offers of IIRs received from healthcare professionals, particularly those with psychosocial problems. However, a number of patients who had overcome their resistance to offers of IIRs appeared to have done so because they felt that diabetes specialist nurses were listening to them. Patients valued the proactive service they received from nurses coupled with their practical and understandable advice. Patients also placed value on a style of communication that appeared to be personal and empathetic. It was this aspect of communication that was described, particularly by patients in low socioeconomic groups, as making the service more accessible:

*It’s when I see somebody else I just don’t feel the same. It’s not the same people what I can talk to, open up to. If it’s (diabetes specialist nurse’s name) I can open up to her and everything. Anybody else I couldn’t do it. I know it sounds a bit daft but it’s what I've got to get used to.*

*(Patient 6 – MDI – Routine and manual – unemployed)*

The practical and empathetic approach adopted by nurses appeared to be of more importance for patients in lower socioeconomic groups who were struggling with glycaemic control. These patients valued the non-judgmental approach adopted by nurses:

*I think she's completely on your level. She respects your views and your opinions compared to other people. So she's just helpful. She’ll sit there and listen to you and support you.*

*(Patient 8 – MDI – Intermediate occupations)*

#### A structured education programme facilitated equitable access

One of the important socioeconomic disparities between patients in this sample related to knowledge of managing diabetes and the use of this knowledge to gain access to IIRs. In this sample an extremely important source of information for patients was the Structured Education Programme. Barriers to accessing the Structured Education Programme for patients in low socioeconomic groups were noted in the earlier theme on permeability. The majority of patients in the sample who had attended the course described its impact as significant in terms of helping them to manage diabetes more effectively and improve their motivation with, for example, undertaking more blood glucose testing. Being able to access the Structured Education Programme enabled some patients to acquire attributes valued by healthcare professionals (higher health literacy levels, motivation and confidence to manage a more complex regimen) and hence appeared to be an important aspect of gaining access to IIRs and ameliorating socioeconomic disparities.

## Discussion

In this study access to an IIR was a two stage process involving ‘access-entry’ and ‘in-system’ access [29]. First, patients needed to gain ‘access-entry’ to specialist services before being offered an IIR. We found that this was influenced by lack of permeable services for patients in low socioeconomic groups. Second, ‘in-system’ access was influenced by the ability of patients to present as candidates for IIR and this in turn was influenced by issues of alignment with healthcare professionals, degree of fit and healthcare professional judgements.

### Why some patients do not access specialist services

We found that lack of permeability affected access for some individuals who had opted for care at their GP practice for reasons of convenience rather than an expressed preference for these services. Issues of permeability also acted as a barrier for those with ‘chaotic’ lives who struggled to attend hospital appointments. This resonates with previous quantitative research linking deprivation with non-attendance at hospital outpatients’ clinics [47]. We also found that factors involving transport and work were linked with permeability and influenced decisions to attend specialist services. The findings in relation to work were in line with previous research which suggested that work commitments were amongst the commonest reasons given for non-attendance at general practices and NHS outpatient clinics [48]. Our study provided a more nuanced understanding of this barrier since it revealed the financial implications for individuals in low skilled and low paid jobs taking time off work for hospital appointments. The findings also resonate with a recent study which found that individuals with type 1 diabetes in the most deprived categories were more frequently disengaged from healthcare services [49] . In our study issues relating to transport impeded some individuals’ attendance at specialist services and these findings are consistent with studies which have found transport is an important barrier to accessing services for lower socioeconomic groups [29].

### Alignment with healthcare professionals

In our study patient perceptions of negative communication with healthcare professionals appeared to affect access to IIR. Some individuals who were dissatisfied with their interactions with healthcare professionals opted out of specialist services. This aligned with the findings of a study exploring reasons for non-attendance by young adults with type 1 diabetes, in which concerns about receiving negative comments regarding failure to achieve target HbA1c levels influenced decisions to attend appointments [50]. Our findings that non-alignment between the patient and the healthcare professional leads to difficulties in patient-provider communication and thus access to IIR is consistent with a number of studies that highlight the style of communication experienced by individuals who have difficulty managing diabetes [50, 51]. We found, like Wikblad, that patients with good metabolic control reported receiving positive responses from healthcare professionals, while those with unsatisfactory HbA1c results felt both coerced and unsupported [52]. Our findings also resonated with the recent finding that some healthcare professionals categorise patients with type 1 diabetes as ‘good’ or ‘bad’ depending on their achievement of acceptable HbA1c levels [53].

### How the ‘degree of fit’ impacts access

In our study having a proactive approach to communication appeared to facilitate access to an IIR because these patients had an ability to engage with healthcare professionals and build rapport which eased the process of accessing an IIR. Patients who described being engaged in consultations appeared to be articulate, self-confident and often from professional backgrounds. They appeared to be able to assert their claim to candidacy. Others have found that ‘ideal’ patients have a good ‘fit’ with the health care services on offer since they have ‘the exact set of competencies and resources required to make optimal use of the service’ [29] (p.53). The concept of the ‘ideal user’ suggests that where there is a match or alignment between patient preferences and services offered by healthcare professionals access to services is easier. Our findings would appear to substantiate the claim by Dixon et al. [54] that the middle classes are able to get a better service than other patients through using their ‘voice’. However, focusing on the ‘ideal’ patient may exclude individuals who for a variety of reasons do not conform to healthcare professional notions of an ‘ideal’ candidate.

### Are healthcare professional judgments biased against some potentially eligible candidates?

In seeking patients suitable for IIRs, in addition to clinical factors, healthcare professionals in our study reported selecting individuals on the basis of evidence of one or more of the following: motivation; potential ability to use IIRs and knowledge. All of these were considered essential to ensure that patients were safe, particularly in relation to CSII. Previous research also suggests judgments about patient suitability for CSII are based on a number of non-clinical factors including personal and psychological attributes in order to decide which patients will make optimum use of CSII [55]. It is interesting that healthcare professionals’ assumptions about patient suitability can be challenged and found to be inaccurate, with some patients unexpectedly going on to have success with CSII [55].

### Diabetes specialist nurses facilitate access to intensive insulin regimens

An important finding was that the services provided by diabetes specialist nurses appeared to facilitate access to IIRs. Diabetes specialist nurses appeared to be in tune with wider aspects of patients’ lives that may impact on their ability to manage diabetes and access IIR. This empathetic approach to communication has been found elsewhere where nurses can broaden their discussion with patients to encompass aspects of lifestyle and other health related issues [56].

### Diabetes structured education Programme minimises disparities in access to an IIR

We found that attending a diabetes Structured Education Programme was a key influence on accessing IIRs. It appeared that some of the characteristics healthcare professionals sought in patients could be attained through education. Attending the course appeared to minimise disparities in ability, knowledge and motivation amongst participants. This appears to be in line with a study in which socioeconomic differences in HbA1c values, amongst patients with both type 1 and type 2 diabetes, were ameliorated by a Structured Education Programme and treatment in specialist services [57]. In our study, acquiring knowledge about diabetes management and alternative regimens seemed to allow individuals to participate more fully in consultations and to manage regimens more effectively. The ability of a Structured Education Programme to empower patients and to lead to a different kind of communication with healthcare professionals resonates with the findings of a longitudinal study conducted with participants of a Dose Adjustment for Normal Eating (DAFNE) course [58].

### Strengths and weaknesses of the current study

The current study set out to elucidate the factors involved in known socioeconomic disparities in access to IIRs. A key strength was to combine both patient and provider perspectives on gaining access to IIRs, providing an in-depth exploration of the complex factors involved. A potential limitation however, was that the study was undertaken in a single specialist diabetes service in the UK and hence this may affect transferability of findings. In addition the majority of participants had achieved access to an IIR. However, it could be argued that an in-depth investigation of this group of participants enabled some key insights into the ways in which individuals had gained access to the technology. An important limitation was that there were fewer individuals from lower socioeconomic backgrounds than desired and hence the study may not have achieved saturation of themes. The exclusion of non-English speaking participants may have also led to selection bias.

Further work could investigate the transferability of these findings to other settings and could focus predominantly on hard to reach groups, non-English speaking individuals and those not currently accessing specialist services. The current study identified health literacy as an influencing factor in relation to gaining access to IIRs. Further research is needed to assess the impact of interventions designed to improve health literacy amongst adults with type 1 diabetes.

### Implications for policy and practice

The current study suggests that lower socioeconomic groups may be at a disadvantage in relation to accessing IIRs compared with their counterparts in higher socioeconomic groups. Commissioners and healthcare professionals involved in designing services could consider ways of improving access to specialist healthcare services for adults with type 1 diabetes who face difficulties due to low paid work or transport. Some of the barriers associated with access for these groups were modifiable through contact with diabetes specialist nurses and the Structured Education Programme; hence access to these aspects of services should be facilitated.

## Conclusions

Access to IIRs appeared to be reduced for people from lower socioeconomic groups by a complex mix of factors relating to patients, their interactions with the healthcare system and patient-provider communication. Ability to access specialist services was influenced by personal social circumstances including low paid work and transport difficulties. Factors diminishing candidacy for IIRs were low health literacy, non-alignment with healthcare professional goals, psychosocial problems and poor quality patient-provider communication. Hence some healthcare professionals’ judgments around suitability for IIRs may inadvertently disadvantage individuals in lower socioeconomic groups. Efforts to promote access to diabetes specialist nurses and a diabetes Structured Education Programme could ameliorate socioeconomic disparities.

## Data Availability

The dataset comprises qualitative interviews with participants and includes information from which participants could be potentially identifiable. For this reason the raw dataset will not be made available due to participant confidentiality restrictions.

## Abbreviations

CSII: Continuous subcutaneous insulin infusion
DAFNE: Dose Adjustment for Normal Eating
HbA1c: Haemoglobin A1c
IIR: Intensive Insulin Regimen
